# Short-Term Efficacy and Safety of Elobixibat for Chronic Constipation Assessed by Rectal Ultrasonography: A Retrospective Observational Study

**DOI:** 10.3390/diagnostics16020354

**Published:** 2026-01-21

**Authors:** Momoko Tsuda, Tomoyuki Onodera, Kanako Konishi, Norishige Maiya, Mio Matsumoto, Kimitoshi Kubo, Sayaka Kudo, Yoshiyuki Hosoi, Mototsugu Kato

**Affiliations:** 1 Department of Gastroenterology, National Hospital Organization Hakodate Medical Center, 18-16, Kawahara-cho, Hakodate 041-8512, Japan; momoko0221tsuda@gmail.com (M.T.);; 2 Department of Gastroenterology, Sapporo Cancer Screening Center, Public Interest Foundation Hokkaido Cancer Society, 1-15, Kita-26 Higashi-14, Higashi-ku, Sapporo 065-0026, Japan; 3 Department of Clinical Laboratory, National Hospital Organization Hakodate Medical Center, 18-16, Kawahara-cho, Hakodate 041-8512, Japan; 4 Department of Laboratory, Sapporo Cancer Screening Center, Public Interest Foundation Hokkaido Cancer Society, 1-15, Kita-26 Higashi-14, Higashi-ku, Sapporo 065-0026, Japan; 5 Medical Department, EA Pharma Co., Ltd., Sumitomo Irifune Bldg, 2-1-1, Irifune, Chuo-ku, Tokyo 104-0042, Japan

**Keywords:** constipation, rectal ultrasonography, elobixibat, ileal bile acid transporter, fecal retention, gas retention, retrospective observational study

## Abstract

**Background/Objectives:** Ultrasonography (US) is a non-invasive and repeatable examination for evaluating chronic constipation. However, few studies have explored treatment decisions based on rectal US findings. To date, the efficacy and safety of elobixibat have not been evaluated using rectal US classification in patients with chronic constipation. This study aimed to evaluate the short-term efficacy and safety of elobixibat in patients with chronic constipation classified as “no fecal retention” by rectal US. **Methods:** We retrospectively analyzed 32 patients with chronic constipation who underwent rectal US and received elobixibat (10 mg/day) between May 2019 and December 2024. Rectal US findings classified patients into four groups: no fecal retention, fecal retention without hard stools, fecal retention with hard stools, and gas retention. The primary endpoint was the response rate of spontaneous bowel movements (SBMs) within 3 days after starting elobixibat in the “no fecal retention” group. **Results:** Among 18 patients in the “no fecal retention” group, 94.4% achieved SBMs within 3 days, indicating a favorable short-term response. Adverse events included abdominal distension and abdominal pain, each observed in one patient (3.1%). **Conclusions:** Elobixibat was effective and well tolerated in patients with chronic constipation classified by rectal US findings.

## 1. Introduction

In Japan, 2.8% of men and 4.4% of women are reported to experience constipation, which significantly impacts quality of life (QOL) [1,2,3]. In routine clinical practice, constipation is often managed without objective or standardized assessment tools, and treatment decisions therefore rely largely on patients’ subjective symptom reports. This dissatisfaction often drives patients to attempt self-management, leading to treatment that increasingly deviates from appropriate care.

Ultrasonography (US) is a non-invasive examination that can be performed repeatedly. Consequently, it has been considered useful for evaluating chronic constipation, which often requires repeated assessment [4,5,6]. Furthermore, the Study Group for US for Chronic Constipation proposed diagnosis and treatment algorithms based on rectal US findings and compiled them into a consensus document [5,7]. The consensus document classifies chronic constipation into three categories (“no fecal retention,” “fecal retention without hard stools,” and “fecal retention with hard stools”) based on rectal US findings and proposes appropriate management for each category.

Elobixibat, an ileal bile acid transporter (IBAT) inhibitor, blocks bile acid reabsorption in the ileum, thereby increasing the amount of bile acids entering the colon [8]. These bile acids increase water and electrolyte influx into the colon, triggering strong bowel movements. In addition, elobixibat lowers rectal sensory thresholds and restores the urge to defecate in patients with chronic constipation [9,10]. Therefore, elobixibat is a promising treatment option for constipation because its mechanism differs from that of conventional laxatives [7,10].

The diagnosis and treatment algorithm for chronic constipation using rectal US findings recommends elobixibat, an IBAT inhibitor, as a first-line treatment for patients with “no fecal retention” whose condition does not improve with lifestyle modification or dietary therapy [7]. This algorithm was developed based on expert opinion as well as drug characteristics. However, few studies have validated this three-category US classification and the corresponding management strategies, indicating the need for further evaluation. Moreover, in real-world practice, the presence of rectal gas cannot be ignored. Rectal evacuation disorder (RED) is a leading cause of intractable constipation not represented in the current three-category classification. Gas retention in the rectum and colon has been reported in patients with RED [11]. In addition, gas retention in the descending colon has also been described among patients reporting incomplete evacuation [12]. These patients may present with features not explained by the three-category classification, indicating that gas-dominant constipation is not well represented in the current system.

This retrospective observational study aimed to evaluate the short-term efficacy and safety of elobixibat in patients with chronic constipation classified as “no fecal retention” by rectal US. Furthermore, the study addressed gas-dominant constipation by expanding the conventional three-category classification to four categories through the addition of “gas retention”: “no fecal retention,” “fecal retention without hard stools,” “fecal retention with hard stools,” and “gas retention.” We retrospectively analyzed patients with chronic constipation who underwent rectal US and received elobixibat and evaluated treatment outcomes according to this four-category classification. This study demonstrates that rectal US-based classification, especially the “no fecal retention” category, provides a practical framework for guiding elobixibat therapy in patients with chronic constipation.

## 2. Materials and Methods

### 2.1. Study Participants

This retrospective, single-arm observational study utilized data collected between May 2019 and December 2024 at Hakodate Medical Center and Sapporo Cancer Screening Center. The study enrolled 32 patients aged ≥18 years with chronic constipation, diagnosed according to Japanese clinical guidelines, who underwent rectal US and received elobixibat [6,13]. Patients who underwent rectal US on the day before the first elobixibat administration and received the drug at the recommended dose of 10 mg/day were included. The exclusion criteria were as follows: a history of hypersensitivity to elobixibat; confirmed or suspected intestinal obstruction caused by tumors, hernia, or other causes; suspected constipation secondary to organic disease; or the use of intestinal cleansing agents, enemas, or bowel lavage from 2 days before to 3 days after rectal US.

### 2.2. Observation Period and Data Collection

Patient data were collected from the day before elobixibat initiation until 2 weeks after treatment. Information was collected from medical records and questionnaires, including age, sex, height, weight, comorbidities, prior medications, rectal US findings, colonic diameters, elobixibat dosing, spontaneous bowel movements (SBMs) during the first 3 days after elobixibat initiation, Bristol Stool Form Scale (BSFS), Constipation Scoring System (CSS), and adverse events [14,15].

### 2.3. Assessment of Bowel Movements

Bowel function was assessed by documenting the presence or absence of SBMs during the first 3 days after elobixibat initiation using patient questionnaires. SBMs were defined as bowel movements occurring without the use of bisacodyl suppositories, enemas, or digital disimpaction. Patients who experienced SBMs within this period were defined as responders.

### 2.4. Stool Form and Constipation Scoring

Stool consistency and constipation severity were evaluated using the BSFS and calculated modified CSS scores, respectively, based on entries in medical records and questionnaires. The modified CSS total score was calculated by subtracting the item “duration of constipation” from the CSS total score. BSFS and CSS were assessed at baseline and 2 weeks after treatment.

### 2.5. Ultrasonography (US)

Rectal ultrasound images were obtained using an Aplio i700^®^ system (Canon Medical Systems, Tochigi, Japan) equipped with a convex probe (PVI-475BX). Imaging conditions were set as follows: depth, 14–21 cm; frequency, 4.0 MHz; gain, 73–80 dB; and dynamic range, 60 dB. Rectal US was performed in the supine position using a transabdominal approach with the probe placed on the suprapubic region. Examinations were conducted by two physicians following previously reported methods [16,17]. A 1-day training session was held before study initiation to standardize the examination technique among the physicians. Rectal US images were assessed by sonographers, who had access to clinical symptoms and prior treatments.

### 2.6. Rectal US Classification

**Figure 1:**
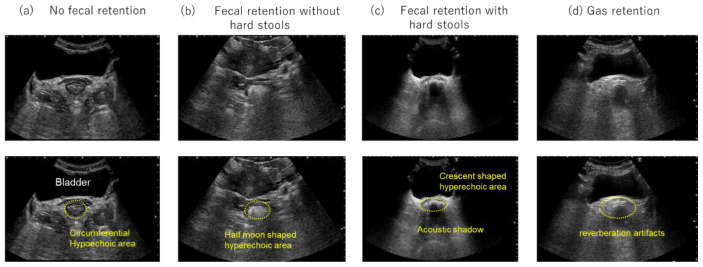
Transverse rectal ultrasound images. (**a**) No fecal retention, with no hyperechoic area observed. A circumferential hypoechoic area is observed in the lower part of the bladder. (**b**) Fecal retention without hard stools, revealing a half-moon-shaped hyperechoic area in the lower part of the bladder. (**c**) Fecal retention with hard stools, revealing a crescent-shaped hyperechoic area with an acoustic shadow in the lower part of the bladder. (**d**) Gas retention, multiple echoes with reverberation artifacts are observed in the lower part of the bladder.

To classify rectal findings, the conventional three-category classification (no fecal retention, fecal retention without hard stools, and fecal retention with hard stools) was expanded by adding a fourth category, “gas retention.” The “gas retention” category was defined as the presence of multiple echoes with reverberation artifacts in the rectum on transabdominal US (Figure 1).

### 2.7. Measurement of Colonic Diameter

To measure colonic diameter, US was performed at five sites: ascending colon, transverse colon, descending colon, sigmoid colon, and rectum [4]. Up to three measurements (proximal, middle, and distal) were obtained for each site. The diameter for each site was calculated as the average of its measurements, and the mean colonic diameter was obtained by averaging across all sites. Colonic diameter was assessed at baseline only.

### 2.8. Statistical Analysis

All analyses were conducted using R (version 4.4.1; R Core Team, Vienna, Austria, 2024). The efficacy analysis was performed using the per-protocol set, which included patients who met all eligibility criteria, did not meet any exclusion criteria, continued treatment until the scheduled assessment, and had available data. The safety analysis set included all patients who received at least one dose of elobixibat. Baseline characteristics were summarized for both analysis sets. Safety outcomes, including adverse events and treatment discontinuations, were evaluated in the safety analysis set, whereas all other analyses were conducted in the efficacy analysis set.

As this was a retrospective study, the sample size was not determined based on statistical power calculations but was set according to the number of patients that could reasonably be collected at the participating sites. Missing data were not imputed; all analyses were based on observed cases. Continuous variables are summarized as means with standard deviations or medians with interquartile ranges, and categorical variables as frequencies and percentages. The Wilson score method was used to calculate 95% confidence intervals (CIs). Before-and-after values were compared using the Wilcoxon signed-rank test. A two-sided *p*-value < 0.05 was considered statistically significant. A correlation analysis was conducted to examine the association between mean colonic diameter and rectal US classification. For this analysis, only patients with measurements available for all five colonic regions were included. We used linear regression models with “no fecal retention” as the reference group to estimate differences in transverse diameter.

## 3. Results

### 3.1. Patient Enrollment and Analysis Sets

**Figure 2:**
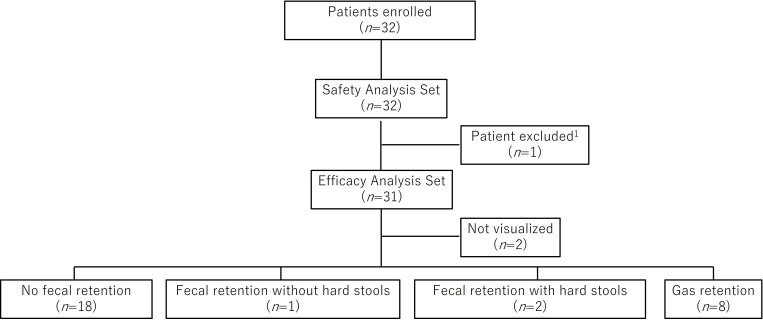
Patient flow diagram. Thirty-two patients were enrolled, of whom 32 were included in the safety analysis set and 31 in the efficacy analysis set. Patients were classified into four rectal US categories: no fecal retention (*n* = 18), fecal retention without hard stools (*n* = 1), fecal retention with hard stools (*n* = 2), and gas retention (*n* = 8). ^1^ One patient with a colostomy was excluded because rectal US classification could not be performed.

During the study period, 249 patients underwent rectal US, and 110 were prescribed elobixibat after the procedure. Among them, 32 patients met the eligibility criteria and were enrolled in the study. All 32 patients were included in the safety analysis set. One patient with a colostomy was excluded from the efficacy analysis set, leaving 31 patients for efficacy evaluation. Based on rectal US findings, 18 patients were classified as “no fecal retention,” 1 as “fecal retention without hard stools,” 2 as “fecal retention with hard stools,” and 8 as “gas retention.” Rectal US findings could not be visualized in two patients (Figure 2).

### 3.2. Patient Characteristics

Patient characteristics are summarized in Table 1. Most patients were women in the efficacy and safety analysis sets. The mean age was 56.8 years in the efficacy set and 56.5 years in the safety set. Baseline bowel movement data (median) for the efficacy and safety sets, respectively, were as follows: BSFS score, 1.0 and 1.0; CSS total score, 13.5 and 13.5; and modified CSS total score, 10.0 and 10.0. CSS sub-scores were as follows: frequency of bowel movements, 1.0 and 1.0; painful evacuation effort, 2.0 and 2.0; feeling of incomplete evacuation, 3.0 and 3.0; abdominal pain, 1.0 and 1.0; minutes in lavatory per attempt, 1.0 and 1.0; type of assistance, 1.0 and 1.0; unsuccessful evacuation attempts per 24 h, 1.0 and 1.0; and duration of constipation, 3.0 and 3.0 (Table 1).

### 3.3. Proportion of Responders with SBMs Within 3 Days After the First Dose of Elobixibat

The primary endpoint was the proportion of responders in the “no fecal retention” category who had SBMs within 3 days after the first dose of elobixibat. In this category, 94.4% (95% CI, 74.2–99.0%) achieved SBMs. Response rates were 100% in the “gas retention” category (95% CI, 67.6–100.0%). The observed response rate across all four categories was 96.6% (95% CI, 82.8–99.4%) (Table 2). Both patients whose rectal US findings were not visualized also responded.

### 3.4. Proportion of Responders with SBMs on Day 1 Following the First Dose of Elobixibat

On day 1, 83.3% (95% CI, 60.8–94.2%) of patients in the “no fecal retention” category achieved SBMs. Response rates were 100% in the “gas retention” category (95% CI, 67.6–100.0%). The observed response rate across all four categories was 89.3% (95% CI, 72.8–96.3%) (Table 3).

### 3.5. Stool Consistency Outcomes (BSFS)

Changes in stool consistency were evaluated using the BSFS. In patients classified as “no fecal retention,” the median BSFS scores were 1.5 (1.0–5.0) at baseline and 4.0 (2.5–5.0) at week 2, showing no significant difference. In contrast, patients in the “gas retention” category demonstrated a significant improvement in BSFS scores from 1.0 (1.0–3.0) at baseline to 4.0 (2.5–4.5) at week 2 (*p* = 0.0350). When analyzed across all four categories, BSFS scores significantly improved from 1.0 (1.0–3.5) at baseline to 4.0 (2.0–5.0) at week 2 (*p* = 0.0104) (Table 4).

**Figure 3:**
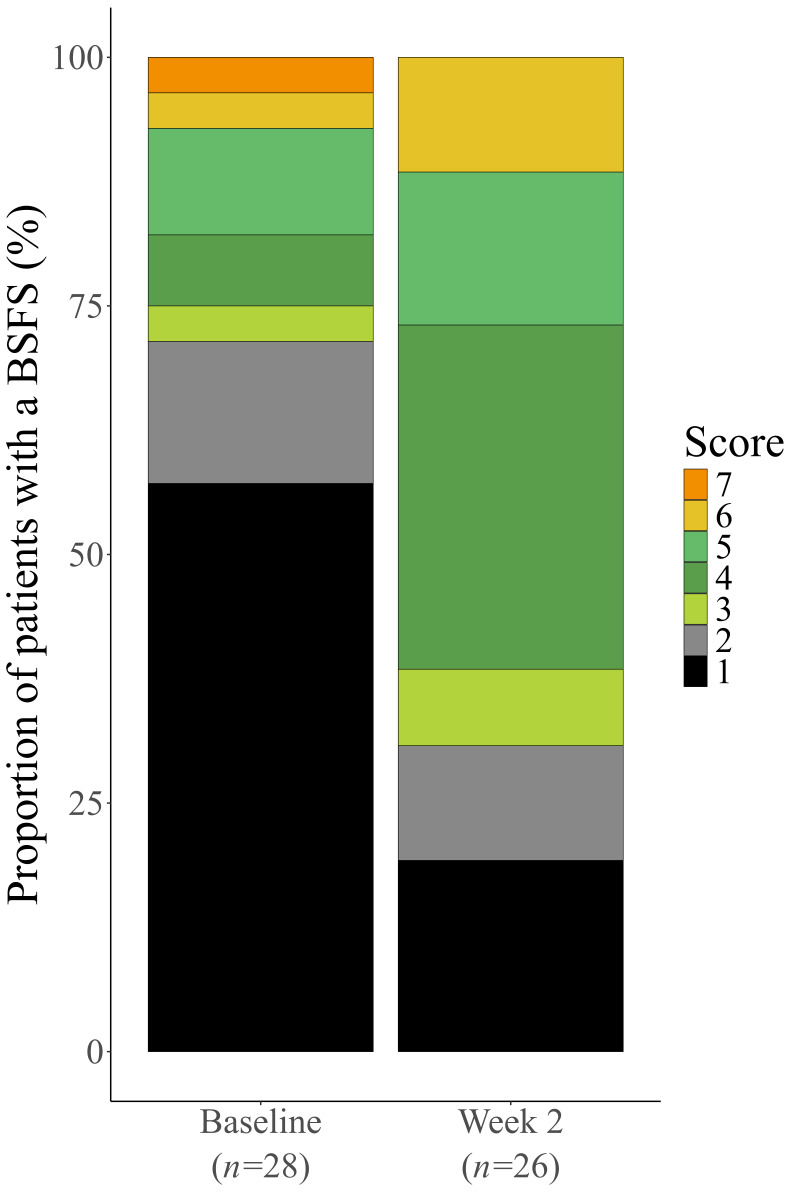
Distribution of stool consistency in all four categories combined. BSFS score definitions: 1 = separate hard lumps, like nuts; 2 = sausage-shaped but lumpy; 3 = like a sausage or snake, but with cracks on its surface; 4 = like a sausage or snake, smooth and soft; 5 = soft blobs with clear-cut edges; 6 = fluffy pieces with ragged edges, a mushy stool; 7 = watery, no solid pieces. Data were compiled excluding patients with “Not evaluated” BSFS scores (baseline, *n* = 1; week 2, *n* = 3).

We also analyzed the distribution of stool consistency in the total cohort. The proportion of patients with a BSFS score of 1 or 2 decreased from 71.4% (20/28 patients) at baseline to 30.8% (8/26 patients) after elobixibat treatment. The proportion of patients with a BSFS score of 3–5 increased from 21.4% (6/28 patients) to 57.7% (15/26 patients), and that of patients with a BSFS score of 6 or 7 increased from 7.1% (2/28 patients) to 11.5% (3/26 patients). These findings indicate that elobixibat shifted stool consistency toward normal or softer stools (Figure 3).

### 3.6. Constipation Scoring System (CSS) Outcomes

#### 3.6.1. Modified CSS Total Score

Changes in the modified CSS total score were evaluated. In patients classified as “no fecal retention,” the median score decreased from 10.0 (7.0–14.0) at baseline to 8.0 (6.0–12.0) at week 2, with no significant difference. In patients classified as “gas retention,” the score decreased from 10.0 (6.0–10.0) at baseline to 6.0 (3.0–11.5) at week 2, but the change was not statistically significant (*p* = 0.0502). When analyzed across all four categories, the modified CSS total score significantly decreased from 10.0 (7.5–13.5) at baseline to 8.5 (5.0–12.0) at week 2 (*p* = 0.0231) (Table 5).

#### 3.6.2. Modified CSS Sub-Scores

Changes in individual CSS sub-scores were also examined. No significant differences were observed in any sub-score when patients were classified by rectal US classification.

**Figure 4:**
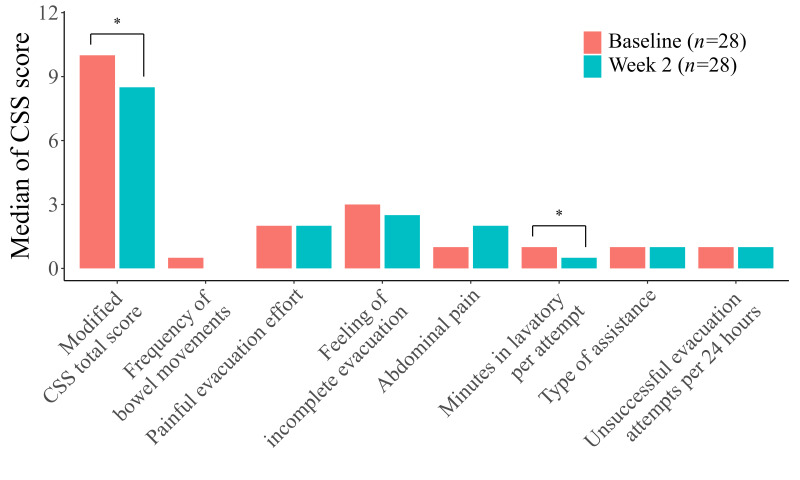
Modified Constipation Scoring System (CSS) sub-scores in all four categories combined. Statistical analyses were performed using the Wilcoxon signed-rank test. Bars indicate items with statistically significant changes. * *p* < 0.05.

However, when analyzed across all four categories, the sub-score “minutes in lavatory per attempt” showed significant improvement after elobixibat treatment (*p* = 0.0046). No other sub-scores demonstrated significant changes (Table 6, Figure 4).

### 3.7. Relationship Between Colonic Diameter and Rectal US Classification

The relationship between the mean transverse colonic diameter and rectal US classification was evaluated. The mean diameter was 25.00 ± 1.23 mm in patients with “no fecal retention,” and 28.86 ± 1.43 mm in those with “gas retention.” The overall mean diameter across all four categories was 27.31 ± 3.41 mm (Table 7). A correlation ratio of 0.694 was observed between the total classification and mean transverse diameter, indicating a positive association (Table S1).

Linear regression analysis showed that the mean transverse diameter was significantly greater in patients with “gas retention” (*p* = 0.0027) than in those without fecal retention (Table 8).

### 3.8. Safety

Adverse events occurred in two patients (6.3%) in the safety analysis set (Table 9). All events were mild and resolved without intervention. No serious adverse events occurred, and no patients discontinued elobixibat during the 2-week observation period.

## 4. Discussion

This retrospective study demonstrated that elobixibat was highly effective and well tolerated in patients with chronic constipation, particularly in those categorized as “no fecal retention” on rectal US. The primary endpoint, defined as the proportion of responders achieving SBMs within 3 days of the first dose, was 94.4% in this group, underscoring the rapid therapeutic benefit of elobixibat. Early symptom relief is clinically relevant because it improves treatment adherence and patient satisfaction. The day 1 response rate (83.3%) was also consistent with previous Phase 3 data (≤24 h: Elobixibat, 86%; Placebo, 41%) [18]. However, these results are provided for supportive context only and should be interpreted carefully, as differences in study design and conditions preclude direct comparison across trials.

The therapeutic benefit in the “no fecal retention” group can be explained by the unique pharmacological properties of elobixibat. Unlike conventional laxatives, elobixibat promotes bowel movements through multiple mechanisms: inhibition of IBAT to increase bile acid flow and fluid secretion, acceleration of large intestinal motility, and restoration of the urge to defecate [9,10]. Kessoku et al. recently reported that, in patients with “no fecal retention,” elobixibat significantly improved complete SBMs, colonic transit time, and the urge to defecate compared with magnesium oxide [19]. Other studies have also shown that elobixibat enables switching from, or reducing the dose of, stimulant laxatives [20,21]. Together, these findings highlight elobixibat’s clinical value as a first-line therapy for patients without fecal retention.

Patients with rectal gas retention also responded favorably to elobixibat, suggesting its potential role in gas-dominant constipation. In this category, all patients achieved SBMs within 3 days. Gas retention has been associated with RED and incomplete evacuation [11,12]. Elobixibat has been reported to improve rectal sensory thresholds [9], suggesting it may be particularly effective for constipation accompanied by gas retention. Although decreased rectal sensation has been proposed as an underlying mechanism [22], it requires confirmation in future studies. In addition, structural defecatory disorders, such as rectocele or megacolon, cannot be excluded by rectal US alone, and complementary diagnostic modalities remain necessary [6]. These results are supportive and hypothesis-generating and should be interpreted with caution. Taken together, these findings suggest that rectal gas retention may represent a distinct and clinically relevant constipation phenotype. Future prospective studies with larger sample sizes are warranted to clarify the pathophysiology of gas-dominant constipation and to determine whether rectal US–based identification of gas retention can guide personalized therapeutic strategies.

In the analysis including all four categories, the BSFS score significantly improved from 1.0 to 4.0 after 2 weeks of elobixibat treatment. A BSFS score of 4 is an important indicator of QOL improvement in patients with chronic constipation [23]. In the present study, improvement in stool form following elobixibat administration suggests a potential contribution to enhanced patient QOL, representing a clinically relevant outcome. Moreover, the modified CSS total score significantly decreased from 10.0 to 8.5, indicating that elobixibat may contribute to reducing defecation-related discomfort. Although improvements in the CSS total score can be observed as early as 2 weeks of treatment [24], other studies have been reported with longer treatment durations. In hemodialysis patients with chronic constipation, CSS improved significantly at 12 weeks, and these improvements were maintained with long-term treatment [25,26].

The impact of rectal US classification was further reflected in colonic diameter measurements. Patients in the “fecal retention with hard stools” and “gas retention” categories had significantly larger transverse colonic diameters than those in the “no fecal retention” group, indicating that hard stool and gas retention are physiologically linked with upstream colonic dilatation. The results are consistent with previous findings suggesting that elobixibat may improve stool and gas distribution, as reflected by colonic diameter measurements [27]. This supports the clinical utility of rectal US as a non-invasive tool for stratifying constipation phenotypes.

Elobixibat was generally safe and well tolerated in this cohort. Only two patients (6.3%) reported mild gastrointestinal adverse events (abdominal distension and abdominal pain), both of which resolved spontaneously without treatment discontinuation. This profile aligns with previous clinical trials, underscoring the suitability of elobixibat for the long-term management of chronic constipation [18,28].

Some limitations of this study should be noted. The retrospective design, small sample size, and short observation period limit the strength of causal inference. Because there was no control or comparator group, potential biases, such as information bias (e.g., reliance on medical records for symptom assessment), cannot be excluded. In addition, as the study population consisted exclusively of outpatients, patients with more advanced physical limitations were less likely to be included, which may have introduced selection bias. The number of eligible patients was markedly reduced because the study included only those who initiated elobixibat the day after rectal US, excluded individuals who had already been receiving the drug, and excluded patients who underwent enema rather than pharmacologic treatment; consequently, the overall sample size was limited to 32 patients. These design constraints should be considered when interpreting the findings. Subgroup sample sizes were particularly small in the “fecal retention without hard stools” and “fecal retention with hard stools” categories. Accordingly, subgroup analyses outside the “no fecal retention” group should be regarded as exploratory and interpreted with caution. Formal inter-observer reliability was not quantitatively assessed in this study. Although all assessors underwent standardized training prior to study initiation and previous studies have shown that US-based assessment of constipation can be performed reliably by trained non-physician healthcare staff [29], future prospective studies should incorporate formal evaluation of inter-observer reliability. Although this study demonstrates meaningful short-term improvements in stool consistency (BSFS) and constipation severity (CSS), the clinical interpretation of these outcomes inherently requires a longer observation period. The observation period was short and focused on early outcomes; thus, long-term improvements in stool consistency, symptom severity, and QOL could not be assessed. Prior studies have demonstrated the sustained efficacy of elobixibat over longer follow-up periods [26,30], and extended follow-up will be necessary to delineate long-term trajectories of stool normalization and symptom fluctuation, as well as their impact on daily functioning or QOL. Moreover, the epidemiological data presented here are specific to the Japanese population, as the study was conducted exclusively in Japan; therefore, generalizability to other populations remains uncertain. Future studies will need to validate these findings in larger, prospective, multicenter cohorts; include extended follow-up to assess long-term outcomes (stool consistency, symptom severity, and QOL) and to fully characterize the symptomatic and functional benefits of elobixibat; incorporate formal quantitative inter-observer reliability testing; and clarify the role of gas-dominant constipation while refining rectal US–based treatment algorithms for broader clinical application.

Elobixibat showed clear and rapid efficacy in patients with chronic constipation, particularly in those with “no fecal retention” on rectal US. Patients with rectal gas retention also benefited, highlighting the value of incorporating this category into diagnostic algorithms. These findings support rectal US-based classification as a practical tool for guiding treatment and confirm elobixibat as an effective first-line option for patients without fecal retention.

## Figures and Tables

**Table 1:** Patient characteristics.

Data	Efficacy Analysis Set		Safety Analysis Set	
	*N*	Variable	*N*	Variable
Subjects	31		32	
Men, *n* (%) ^1^		6 (19.4)		6 (18.8)
Women, *n* (%) ^1^		25 (80.6)		26 (81.3)
Age (years), mean ± SD	31	56.8 ± 14.7	32	56.5 ± 14.6
Height (cm), mean ± SD	31	157.68 ± 7.19	32	157.56 ± 7.10
Weight (kg), mean ± SD	31	55.51 ± 11.96	32	55.18 ± 11.91
Rectal US classification	31		32	
No fecal retention, *n* (%) ^1^		18 (58.1)		18 (56.3)
Fecal retention without hard stools, *n* (%) ^1^		1 (3.2)		1 (3.1)
Fecal retention with hard stools, *n* (%) ^1^		2 (6.5)		2 (6.3)
Gas retention, *n* (%) ^1^		8 (25.8)		8 (25.0)
Not visualized, *n* (%) ^1,2^		2 (6.5)		3 (9.4)
BSFS, median (IQR)	30	1.0 (1.0–3.0)	31	1.0 (1.0–4.0)
CSS				
Frequency of bowel movements, median (IQR)	30	1.0 (0.0–2.0)	31	1.0 (0.0–2.0)
Painful evacuation effort, median (IQR)	30	2.0 (0.0–3.0)	31	2.0 (0.0–3.0)
Feeling of incomplete evacuation, median (IQR)	30	3.0 (2.0–4.0)	31	3.0 (2.0–4.0)
Abdominal pain, median (IQR)	30	1.0 (0.0–2.0)	31	1.0 (0.0–2.0)
Minutes in lavatory per attempt, median (IQR)	30	1.0 (1.0–2.0)	30	1.0 (1.0–2.0)
Type of assistance, median (IQR)	30	1.0 (1.0–2.0)	31	1.0 (1.0–2.0)
Unsuccessful evacuation attempts per 24 h, median (IQR)	30	1.0 (1.0–1.0)	30	1.0 (1.0–1.0)
Duration of constipation, median (IQR)	30	3.0 (2.0–4.0)	31	3.0 (2.0–4.0)
CSS total score, median (IQR)	30	13.5 (10.0–17.0)	30	13.5 (10.0–17.0)
Modified CSS total score, median (IQR)	30	10.0 (8.0–13.0)	30	10.0 (8.0–13.0)
Colonic diameter				
Rectum (mm), mean ± SD	20	34.15 ± 13.18	20	34.15 ± 13.18
Ascending colon (mm), mean ± SD	24	35.70 ± 6.83	25	35.58 ± 6.72
Transverse colon (mm), mean ± SD	20	24.29 ± 3.34	21	24.35 ± 3.26
Descending colon (mm), mean ± SD	22	22.49 ± 4.02	23	22.26 ± 4.08
Sigmoid colon (mm), mean ± SD	22	21.80 ± 3.49	23	21.71 ± 3.43
Mean colonic diameter (mm), mean ± SD	24	28.32 ± 6.36	25	28.14 ± 6.29
Comorbidities ^3^	31		32	
Yes, *n* (%) ^1^		13 (41.9)		14 (43.8)
Mental disorder, *n* (%) ^1^		3 (9.7)		3 (9.4)
After cancer surgery, *n* (%) ^1^		2 (6.5)		3 (9.4)
Cholecystectomy, *n* (%) ^1^		1 (3.2)		1 (3.1)
Others, *n* (%) ^1,4^		8 (25.8)		8 (25.0)
No, *n* (%) ^1^		18 (58.1)		18 (56.3)
Prior medications for constipation ^3,5^	31		32	
Yes, *n* (%) ^1^		22 (71.0)		23 (71.9)
Osmotic laxatives, *n* (%) ^1^		12 (38.7)		12 (37.5)
Stimulant laxatives, *n* (%) ^1^		13 (41.9)		14 (43.8)
Intestinal secretagogues, *n* (%) ^1,6^		2 (6.5)		2 (6.3)
Others, *n* (%) ^1,7^		4 (12.9)		4 (12.5)
No, *n* (%) ^1^		9 (29.0)		9 (28.1)
Prior medications for other comorbidities ^5^	31		32	
Yes, *n* (%) ^1^		17 (54.8)		18 (56.3)
No, *n* (%) ^1^		14 (45.2)		14 (43.8)

^1^ Percentages are calculated based on the number of patients in each analysis set. ^2^ Includes one patient excluded from the efficacy analysis set. ^3^ Includes duplicate entries. ^4^ Includes anemia, endometriosis, gastroesophageal reflux disease, hypothyroidism, intracranial aneurysm, Ménière’s disease, pneumothorax, uterine leiomyoma, and endolymphatic hydrops, each in one patient. ^5^ Indicates whether any medication was used within 2 weeks prior to the first dose of elobixibat. ^6^ Includes lubiprostone and linaclotide, each in one patient. ^7^ Includes Chinese traditional or herbal medicines in three patients, and enemas and probiotics in one patient each. One patient experienced both gastroesophageal reflux disease and intracranial aneurysm. One patient had received Chinese traditional or herbal medicines and probiotics. Abbreviations: BSFS, Bristol Stool Form Scale; CSS, Constipation Scoring System; US, ultrasonography.

**Table 2:** Proportion of responders with spontaneous bowel movements (SBMs) within 3 days after the first dose of elobixibat.

Rectal US Classification	*N*	Responder, (*n*)	Non-Responder, (*n*)	Proportion of Responders ^1,2^ (%)	95% CI ^3^ (%)
No fecal retention	18	17	1	94.4	74.2–99.0
Fecal retention without hard stools	1	1	0	100.0	20.7–100.0
Fecal retention with hard stools	2	2	0	100.0	34.2–100.0
Gas retention	8	8	0	100.0	67.6–100.0
Total of 4 categories	29	28	1	96.6	82.8–99.4

^1^ Responders were defined as patients who had at least one SBM within 3 days after the first dose of elobixibat without the use of suppositories (e.g., bisacodyl), enemas, or digital disimpaction. ^2^ Values are expressed as percentages based on the number of patients in each rectal US classification. ^3^ CIs were calculated using the Wilson score method. Abbreviation: CI, confidence interval.

**Table 3:** Proportion of responders with spontaneous bowel movements (SBMs) on day 1 following the first dose of elobixibat.

Rectal US Classification	*N*	Responder, (*n*)	Non-Responder, (*n*)	Proportion of Responders ^1,2^ (%)	95% CI ^3^ (%)
No fecal retention	18	15	3	83.3	60.8–94.2
Fecal retention without hard stools	0	0	0	-	-
Fecal retention with hard stools	2	2	0	100.0	34.2–100.0
Gas retention	8	8	0	100.0	67.6–100.0
Total of 4 categories	28	25	3	89.3	72.8–96.3

^1^ Responders were defined as patients who had at least one SBM on day 1 without the use of suppositories (e.g., bisacodyl), enemas, or digital disimpaction. ^2^ Values are expressed as percentages based on the number of patients in each rectal US classification. ^3^ CIs were calculated using the Wilson score method.

**Table 4:** Stool consistency outcomes (BSFS).

Rectal US Classification	Baseline		Week 2		
	*N*	Variable	*N*	Variable	*p* ^1^
No fecal retention, median (IQR)	18	1.5 (1.0–5.0)	16	4.0 (2.5–5.0)	0.1078
Fecal retention without hard stools, median (IQR)	1	1.0 (1.0–1.0)	0	-	-
Fecal retention with hard stools, median (IQR)	2	1.5 (1.0–2.0)	2	1.0 (1.0–1.0)	1.0000
Gas retention, median (IQR)	7	1.0 (1.0–3.0)	8	4.0 (2.5–4.5)	0.0350 *
Total of 4 categories, median (IQR)	28	1.0 (1.0–3.5)	26	4.0 (2.0–5.0)	0.0104 *

* *p* < 0.05. ^1^ Wilcoxon signed-rank test.

**Table 5:** Modified Constipation Scoring System (CSS) total score.

Rectal US Classification	Baseline		Week 2		
	*N*	Variable	*N*	Variable	*p* ^1^
No fecal retention, median (IQR)	18	10.0 (7.0–14.0)	17	8.0 (6.0–12.0)	0.2206
Fecal retention without hard stools, median (IQR)	1	10.0 (10.0–10.0)	1	11.0 (11.0–11.0)	1.0000
Fecal retention with hard stools, median (IQR)	2	16.5 (13.0–20.0)	2	11.5 (11.0–12.0)	0.3711
Gas retention, median (IQR)	7	10.0 (6.0–10.0)	8	6.0 (3.0–11.5)	0.0502
Total of 4 categories, median (IQR)	28	10.0 (7.5–13.5)	28	8.5 (5.0–12.0)	0.0231 *

* *p* < 0.05. ^1^ Wilcoxon signed-rank test.

**Table 6:** Modified Constipation Scoring System (CSS) sub-scores.

Rectal US Classification	CSS Sub-Score	Timepoint	*N*	Variable, Median (IQR)	*p* ^1^
Total of 4 categories	Total score (excluding the duration of constipation) ^2^	Baseline	28	10.0 (7.5–13.5)	0.0231 *
		Week 2	28	8.5 (5.0–12.0)	
	Frequency of bowel movements	Baseline	28	0.5 (0.0–1.5)	0.3543
		Week 2	28	0.0 (0.0–1.0)	
	Painful evacuation effort	Baseline	28	2.0 (0.0–3.0)	0.6806
		Week 2	28	2.0 (0.0–3.0)	
	Feeling of incomplete evacuation	Baseline	28	3.0 (2.0–4.0)	0.0615
		Week 2	28	2.5 (1.0–3.0)	
	Abdominal pain	Baseline	28	1.0 (0.0–2.0)	0.1078
		Week 2	28	2.0 (0.5–2.0)	
	Minutes in lavatory per attempt	Baseline	28	1.0 (0.5–2.0)	0.0046 *
		Week 2	28	0.5 (0.0–1.0)	
	Type of assistance	Baseline	28	1.0 (1.0–2.0)	0.0782
		Week 2	28	1.0 (0.0–1.0)	
	Unsuccessful evacuation attempts per 24 h	Baseline	28	1.0 (1.0–1.0)	0.0519
		Week 2	28	1.0 (0.5–1.0)	

* *p* < 0.05. ^1^ Wilcoxon signed-rank test. ^2^ Modified CSS.

**Table 7:** Mean transverse colonic diameter stratified by rectal ultrasonography (US) classification.

Rectal US Classification	Number of Patients	Mean Transverse Diameter of the Colon and Rectum ^1^ (mm)
	*N* ^2^	Variable
No fecal retention, Mean ± SD	9	25.00 ± 1.23
Fecal retention without hard stools, Mean ± SD	-	-
Fecal retention with hard stools, Mean ± SD	2	33.09 ± 5.88
Gas retention, Mean ± SD	6	28.86 ± 1.43
Total of 4 categories, Mean ± SD	17	27.31 ± 3.41

^1^ The mean transverse colonic diameter is presented as mean ± SD. ^2^ Number of patients in whom all five regions (ascending colon, transverse colon, descending colon, sigmoid colon, and rectum) were successfully measured.

**Table 8:** Prediction of transverse colonic diameter by rectal US classification (linear regression analysis).

Factor	Estimated Difference ^1^	95% CI	*p*
Rectal US classification: fecal retention without hard stools	-	-	-
Rectal US classification: fecal retention with hard stools	8.09	[4.705–11.468]	0.0002 *
Rectal US classification: gas retention	3.86	[1.579–6.139]	0.0027 *

* *p* < 0.05. ^1^ Estimated difference determined by subtracting the “no fecal retention” group from each respective category.

**Table 9:** Adverse events in the safety analysis set.

			Total	Severity		
				Mild	Moderate	Severe
			*n* (%)	*n* (%)	*n* (%)	*n* (%)
Number			32	32	32	32
Adverse event			2 (6.3)	2 (6.3)	0 (0.0)	0 (0.0)
	Gastrointestinal disorders		2 (6.3)	2 (6.3)	0 (0.0)	0 (0.0)
		Abdominal distension	1 (3.1)	1 (3.1)	0 (0.0)	0 (0.0)
		Abdominal pain	1 (3.1)	1 (3.1)	0 (0.0)	0 (0.0)

Adverse events are expressed as percentages of the total number of patients in the safety analysis set. MedDRA/J version 27.1. Abbreviations: MedDRA/J, Medical Dictionary for Regulatory Activities/J.

## Data Availability

The data presented in this study are available on request from the corresponding author. The data are not publicly available due to privacy and ethical restrictions.

## Supplementary Materials

The following supporting information can be downloaded at: https://www.mdpi.com/article/10.3390/diagnostics16020354/s1, Table S1: Correlation analysis between mean transverse colonic diameter ultrasonography (US) diagnostic classification.

## Abbreviations

The following abbreviations are used in this manuscript:

BSFS	Bristol stool form scale
CSS	Constipation scoring system
CI	Confidence Interval
IBAT	Ileal bile acid transporter
MedDRA/J	Medical Dictionary for Regulatory Activities/J
QOL	Quality of life
RED	Rectal evacuation disorder
SBM	Spontaneous bowel movement
US	Ultrasonography
