# Supplementary material for: Short-Term Efficacy and Safety of Elobixibat for Chronic Constipation Assessed by Rectal Ultrasonography: A Retrospective Observational Study
Source: Diagnostics (Basel). 2026 Jan 21;16(2):354. doi: 10.3390/diagnostics16020354 (PMC12839922; doi:10.3390/diagnostics16020354)
Supplement: Supplementary file 1 [file diagnostics-16-00354-s001.zip › diagnostics-4100331-Table S1.pdf]

diagnostics

Article

Short-Term Efficacy and Safety of Elobixibat for Chronic Constipation Assessed by Rectal Ultrasonography: A Retrospective Observational Study

Momoko Tsuda [1,2], Tomoyuki Onodera[3], Kanako Konishi[4], Norishige Maiya[1], Mio Matsumoto[2], Kimitoshi Kubo[1], Sayaka Kudo[5], Yoshiyuki Hosoi[5] and Mototsugu Kato[2,*]

[1] Department of Gastroenterology, National Hospital Organization Hakodate Medical Center, 18-16, Kawahara-cho, Hakodate 041-8512, Japan; momoko0221tsuda@gmail.com (M. T.); nmaiya.ks3@gmail.com (N. M.); kubotti25@yahoo.co.jp (K. K.)

[2] Department of Gastroenterology, Sapporo Cancer Screening Center, Public Interest Foundation Hokkaido Cancer Society, 1-15, Kita-26 Higashi-14, Higashi-ku, Sapporo 065-0026, Japan; kirennjai@yahoo.co.jp (M. M.); mkato1957@gmail.com (M. K.).

[3] Department of Clinical Laboratory, National Hospital Organization Hakodate Medical Center, 18-16, Kawahara-cho, Hakodate 041-8512, Japan; onodera.tomoyuki.rx@mail.hosp.go.jp

[4] Department of Laboratory, Sapporo Cancer Screening Center, Public Interest Foundation Hokkaido Cancer Society, 1-15, Kita-26 Higashi-14, Higashi-ku, Sapporo 065-0026, Japan; kensaka@hokkaido-taigan.jp

[5] Medical Department, EA Pharma Co., Ltd., Sumitomo Irifune Bldg, 2-1-1, Irifune, Chuo-ku, Tokyo 104-0042, Japan; sayaka_kudo@eapharma.co.jp (S. K.); yoshiyuki_hosoi@eapharma.co.jp (Y. H.)

[*] Correspondence: mkato1957@gmail.com; Tel.: +81-11-748-5511.

Supplementary Materials:

Table S1. Correlation analysis between mean transverse colonic diameter ultrasonography (US) classification

	**Mean transverse colonic diameter [1]**
	**Correlation ratio**
Rectal US classification[2]	0.694

[1] The mean transverse colonic diameter was calculated by dividing the sum of the transverse diameters of the measured regions by the number of regions measured.

[2] The rectal US classification comprises the following four categories: “no fecal retention,” “fecal retention without hard stools,” “fecal retention with hard stools,” and “gas retention.”

Abbreviations: US, ultrasonography.
